# Antibody-Free Magnetic Cell Sorting of Genetically Modified Primary Human CD4+ T Cells by One-Step Streptavidin Affinity Purification

**DOI:** 10.1371/journal.pone.0111437

**Published:** 2014-10-31

**Authors:** Nicholas J. Matheson, Andrew A. Peden, Paul J. Lehner

**Affiliations:** 1 Cambridge Institute for Medical Research, University of Cambridge, Hills Road, Cambridge, United Kingdom; 2 Department of Biomedical Science, University of Sheffield, Western Bank, Sheffield, United Kingdom; University of Alberta, Canada

## Abstract

Existing methods for phenotypic selection of genetically modified mammalian cells suffer disadvantages of time, cost and scalability and, where antibodies are used to bind exogenous cell surface markers for magnetic selection, typically yield cells coated with antibody-antigen complexes and beads. To overcome these limitations we have developed a method termed Antibody-Free Magnetic Cell Sorting in which the 38 amino acid Streptavidin Binding Peptide (SBP) is displayed at the cell surface by the truncated Low Affinity Nerve Growth Receptor (LNGFRF) and used as an affinity tag for one-step selection with streptavidin-conjugated magnetic beads. Cells are released through competition with the naturally occurring vitamin biotin, free of either beads or antibody-antigen complexes and ready for culture or use in downstream applications. Antibody-Free Magnetic Cell Sorting is a rapid, cost-effective, scalable method of magnetic selection applicable to either viral transduction or transient transfection of cell lines or primary cells. We have optimised the system for enrichment of primary human CD4+ T cells expressing shRNAs and exogenous genes of interest to purities of >99%, and used it to isolate cells following Clustered Regularly Interspaced Short Palindromic Repeats (CRISPR)/Cas9 genome editing.

## Introduction

Pure populations of transfected or transduced mammalian cells are commonly isolated from mixed samples by co-expression of the gene or shRNA of interest with three sorts of phenotypic marker: an exogenous gene encoding drug or antibiotic resistance; an internal fluorescent protein, such as GFP, enabling Fluorescence-Activated Cell Sorting (FACS); or a cell surface protein combined with antibody labelling. Where antibody labelling of a cell surface marker is used, antibodies may be either conjugated to a fluorochrome for FACS, or to biotin for affinity purification using a solid streptavidin-conjugated matrix, typically magnetic beads [1]. Compared with FACS, immunomagnetic selection is relatively fast, simple and scalable for simultaneous processing of multiple samples and large cell numbers [1], [2]. It is supported by a number of widely used commercial systems [3], [4] including specific product lines for the enrichment of cells using exogenous CD4, H-2k or LNGFR (MACSelect; Miltenyi) or a membrane-targeted mCherry fusion protein (CherryPicker; Clontech) as the cell surface marker for antibody labelling.

Following immunomagnetic selection, cells typically remain coated with magnetic beads and antibody-antigen complexes, risking alteration of their behaviour or viability through cross-linking of cell-surface receptors (triggering signalling) or internalisation of the ferrous beads (leading to toxicity) [5], [6], [7], [8]. Methods have therefore been devised to release the beads through use of a low affinity biotin, cleavage of a nucleic acid linker, or competition with a selected Fab (antigen-binding) antibody fragment [4]. These approaches are limited, however, by requirements for additional individualised reagents and/or leave cells coated with residual antibody-antigen complexes.

**Figure 1:**
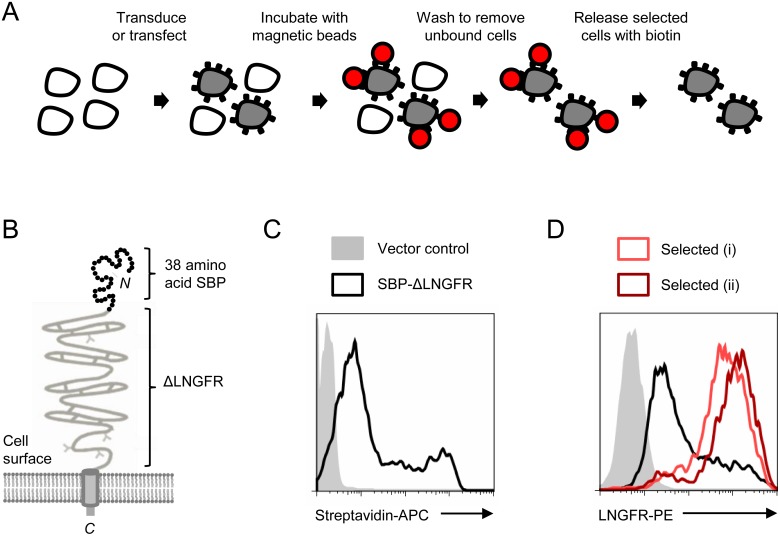
SBP-ΔLNGFR cell surface affinity tag for Antibody-Free Magnetic Cell Sorting. In Antibody-Free Magnetic Cell Sorting (A) transfected or transduced cells co-express a gene or shRNA of interest with a streptavidin-binding cell surface affinity tag. Cells are selected by incubation with streptavidin-conjugated beads then, after washing to remove unbound cells, released by incubation with excess biotin. SBP-ΔLNGFR comprises the 38 amino acid SBP fused to the N-terminus of the truncated LNGFR (B). Expression of SBP-ΔLNGFR at the cell surface was tested 48 hrs after transient transfection of 293Ts with pHRSIN-HA-SBP-ΔLNGFR by staining with streptavidin-APC (C). After a further 72 hrs, cells expressing SBP-ΔLNGFR were selected from the bulk population using magnetic streptavidin-conjugated beads: (i) Dynabeads Biotin Binder (Invitrogen) or (ii) Streptavidin MicroBeads (Miltenyi) (D). Purity of transfected cells before (black) and after (red) selection was assessed by staining with anti-LNGFR-PE. Background staining of cells transfected with a control vector is shown (grey).

Streptavidin-binding peptide tags with nanomolar dissociation constants for streptavidin have been generated for the purification of recombinant proteins [9], [10], [11]. We reasoned that expression of a cell surface streptavidin-binding peptide tag could be used to select cells co-expressing a gene or shRNA of interest by binding directly to streptavidin beads, without the need for antibody labelling. Furthermore, selected cells could subsequently be released from the beads by incubation with biotin, a naturally occurring vitamin already present in many cell culture media, leaving cells free of antibody and beads (Figure 1A). In this report we demonstrate the feasibility of this approach, which we term Antibody-Free Magnetic Cell Sorting, and show that it can be used to obtain genetically modified primary human CD4+ T cells at a purity of >99%. Finally, we adapt the technique for the enrichment of cells following CRISPR/Cas9 genome editing.

## Materials and Methods

### Ethics statement

Ethical permission for this project was granted by the Cambridgeshire 2 Research Ethics Committee (REC reference 97/092). Informed written consent was obtained from all of the volunteers included in this study prior to providing blood samples.

### Antibodies and reagents

The following fluorescent conjugates were used for flow cytometry: ME20.4 anti-LNGFR-PE/APC (BioLegend); BB7.2 anti-HLA-A2-PE (BioLegend); W6/32 anti-MHC-I-AF647 (BioLegend); FN50 anti-CD69-APC (BioLegend); and streptavidin-APC (eBioscience). Bovine Serum Albumin (BSA) Cohn fraction V (A4503; Sigma) which does not contain free biotin was used for Antibody-Free Magnetic Cell Sorting.

### Cell culture

HEK 293 T cells (293Ts) were cultured in DMEM supplemented with 10% FCS and 1% penicillin/streptomycin. Primary human CD4+ T cells were isolated from peripheral blood by density gradient centrifugation using Lympholyte-H (Cedarlane Laboratories) followed by negative selection with the Dynabeads Untouched Human CD4 T Cells Kit (Invitrogen) according to the manufacturer’s instructions. Cells were cultured in RPMI-1640 supplemented with 10% FCS and 1% penicillin/streptomycin and activated within 48 hrs using Dynabeads Human T-Activator CD3/CD28 beads (Invitrogen) according to the manufacturer’s instructions. Purity was assessed by flow cytometry for CD3 and CD4 and typically found to be ≥95%.

### Plasmids

The lentiviral expression construct pHRSIN-HA-HLA-A2 (encoding HLA-A2 with an N-terminal HA tag and a murine immunoglobulin signal peptide) has been previously described [12]. Overlapping DNA oligomers encoding the 38 amino acid SBP [10], [11] were synthesised (Sigma), ligated and inserted using EcoRI/XhoI sites to generate pHRSIN-HA-SBP-HLA-A2. The truncated LNGFR was then amplified by PCR from the retroviral vector pZLRS-IRES-ΔLNGFR [13] and inserted using XhoI/NotI sites in place of HLA-A2 to generate the pHRSIN-HA-SBP-ΔLNGFR construct utilised for pilot experiments in 293Ts (Figure 1).

**Figure 2:**
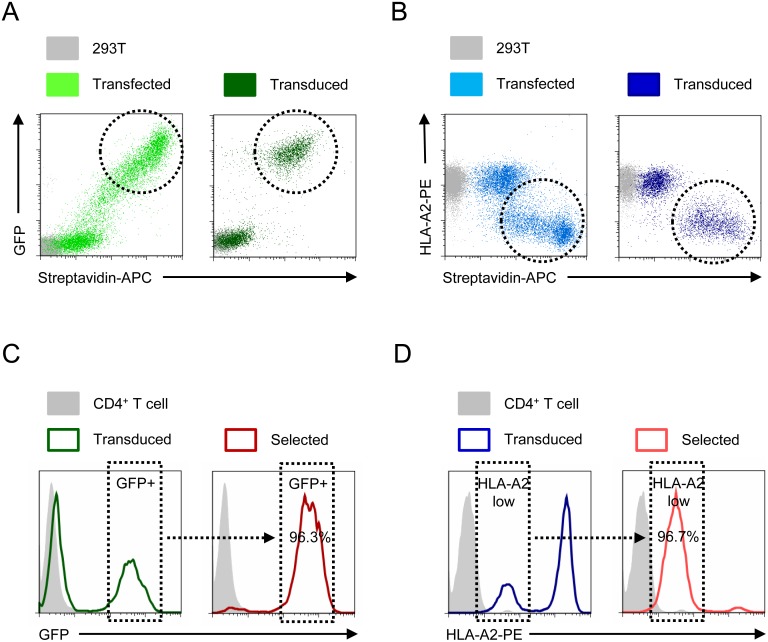
Phenotypic selection using SBP-ΔLNGFR. 293Ts were transiently transfected or lentivirally transduced with pHRSIN-SE-PGK-SBP-ΔLNGFR-W (encoding EGFP and SBP-ΔLNGFR; A) or pHRSIREN/β2 m-PGK-SBP-ΔLNGFR-W (encoding shRNA to β2 m and SBP-ΔLNGFR; B) and stained with streptavidin-APC plus/minus anti-HLA-A2-PE. Transfected/transduced cells are either GFP+/streptavidin-APC+ or HLA-A2-low/streptavidin-APC+ (dashed circles). Primary human CD4+ T cells were lentivirally transduced with the same constructs then selected using Dynabeads Biotin Binder. Purity of cells before (green or blue) and after (red) selection was assessed by GFP fluorescence (C) or staining with anti-HLA-A2-PE (D). Transduced cells are either GFP+ or HLA-A2-low (dashed boxes). Background staining of untransfected/unstransduced controls is shown (grey).

**Figure 3:**
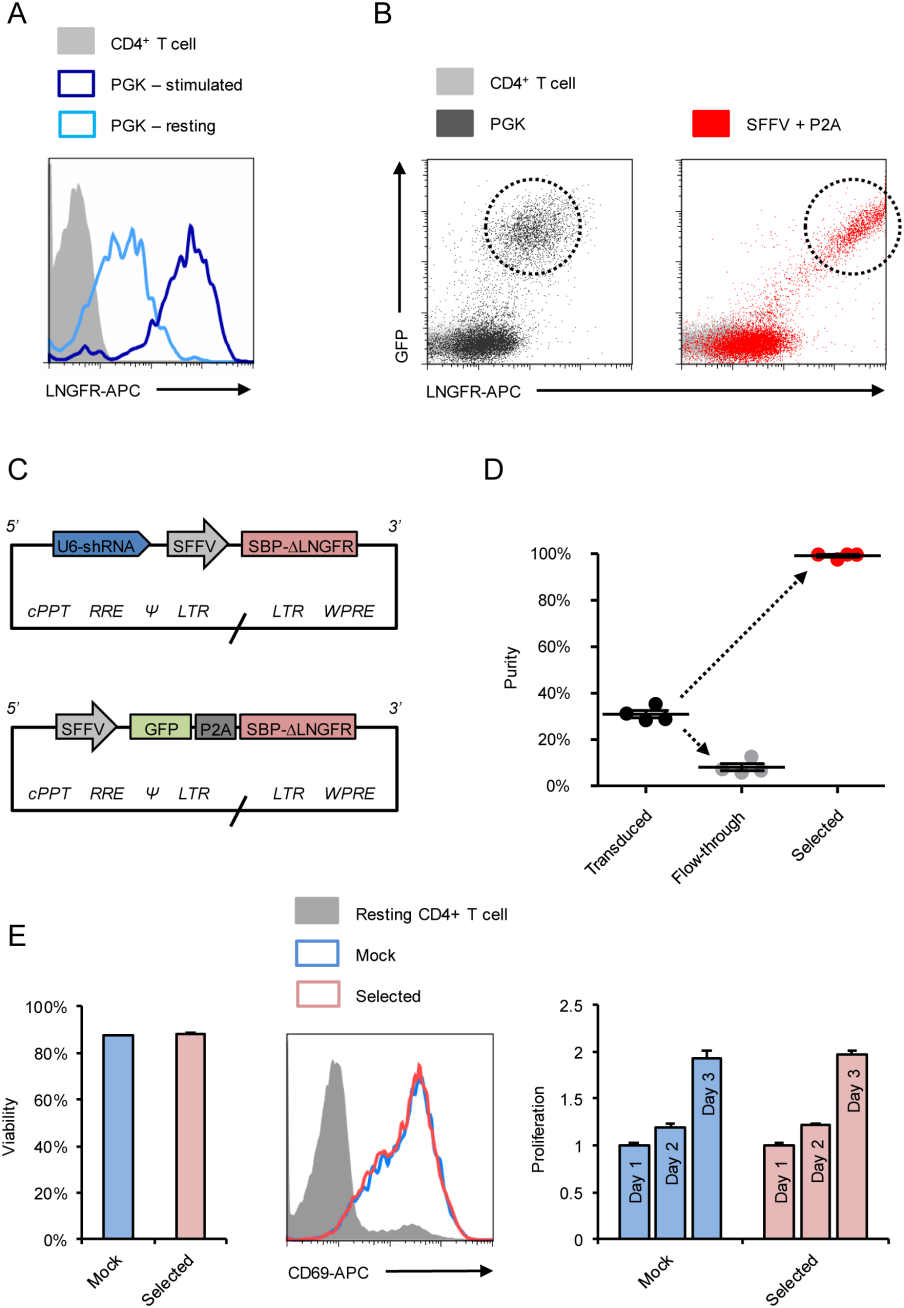
Optimised Antibody Free Magnetic Cell Sorting of primary human CD4+ T cells. Primary human CD4+ T cells were lentivirally transduced with pHRSIREN/β2 m-PGK-SBP-ΔLNGFR-W (encoding shRNA to β2 m and SBP-ΔLNGFR under the PGK promoter) and either rested for 2 weeks (pale blue) or re-stimulated with CD3/CD28 Dynabeads 3 days prior to analysis (dark blue). Cells were co-stained with anti-HLA-A2-PE and anti-LNGFR-APC, and expression levels of SBP-ΔLNGFR compared in HLA-A2-low cells (A). Transduction with pHRSIN-SE-PGK-SBP-ΔLNGFR-W was then compared with pHRSIN-SE-P2A-SBP-ΔLNGFR-W (encoding GFP-P2A-SBP-ΔLNGFR under the SFFV promoter; B). Transduced cells are GFP+/LNGFR-APC+ (dashed circles). Background staining of untransfected/unstransduced controls is shown (grey). Finally, primary human CD4+ T cells were transduced with the optimised pHRSIREN-S-SBP-ΔLNGFR-W and pHRSIN-SE-P2A-SBP-ΔLNGFR-W lentivectors (C) encoding 2 different shRNAs and 2 different exogenous genes. Following selection with Dynabeads Biotin Binder, purity was assessed by staining with anti-LNGFR-PE (D). Each datapoint represents % LNGFR+ for a different construct (shRNA or exogenous gene) and means and SEMs are shown. Viability and functional activity of selected (expressing a control shRNA) and mock (unselected) cells were compared (E). Viability was measured 4 days after selection, and cells either rested or re-stimulated with CD3/CD28 Dynabeads. Resting and re-stimulated cells were stained with CD69-APC (day 2) and enumerated using CytoCount beads (days 1–3). CD69 expression by resting (grey) versus re-stimulated mock (pale blue) and selected (pink) cells is shown. Fold-increases in viable cell numbers following re-stimulation (proliferation) were calculated using day 1 as a baseline. Experiments were conducted in triplicate and means and SEMs are shown. cPPT – central polypurine tract; RRE – Rev response element; * – packaging signal; LTR – long terminal repeat; WPRE – Woodchuck Hepatitis Virus post-transcriptional regulatory element.

**Figure 4:**
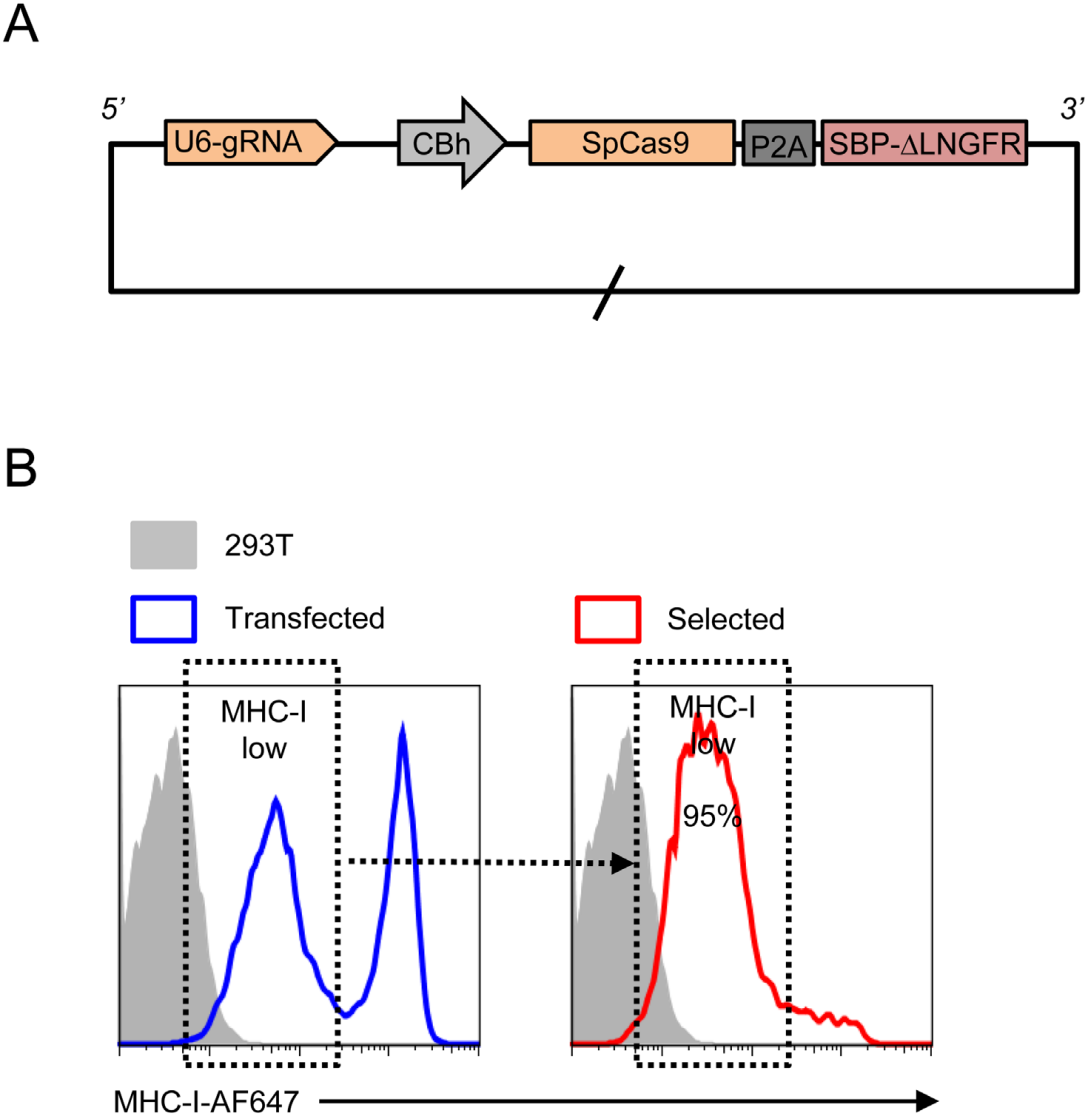
Antibody-Free Magnetic Cell Sorting of 293 T cells following CRISPR/Cas9 genome editing. 293Ts were transiently transfected with pSpCas9(BB)-P2A-SBP-ΔLNGFR (encoding gRNA to β2 m and Cas9-P2A-SBP-ΔLNGFR under the CBh promoter; A) and stained with anti-MHC-I-AF647 before (blue) or after (red) selection with Dynabeads Biotin Binder (B). Transfected cells with β2 m knockouts are MHC-I low (dashed boxes). Background staining of untransfected controls is shown (light grey).

To generate bicistronic lentiviral vectors (Figures 2–4), a codon-optimised SBP-ΔLNGFR fusion protein construct was synthesised in pUC57 (Genscript). For co-expression with an exogenous gene of interest, this construct was subcloned into a self-inactivating lentiviral vector derived from pHRSIN-cPPT-SEW kindly provided by Yasuhiro Ikeda [14] to generate pHRSIN-SE-PGK-SBP-ΔLNGFR-W (encoding SFFV-EGFP and PGK-SBP-ΔLNGFR with a distal WPRE). The PGK promoter was replaced with a *Porcine teschovirus-1* 2A (P2A) sequence [15] synthesised in pUC57 (Genscript) to generate pHRSIN-SE-P2A-SBP-ΔLNGFR-W. BamHI and NotI sites flanking EGFP allow substitution of alternative Genes Of Interest for cotranslation as GOI-P2A-SBP-ΔLNGFR.

For co-expression with an shRNA of interest, the SBP-ΔLNGFR construct was subcloned into a self-inactivating lentiviral vector derived from pCSRQ kindly provided by Greg Towers [16] to generate pHRSIREN-PGK-SBP-ΔLNGFR-W (encoding a U6-shRNA cassette and PGK-SBP-ΔLNGFR with a distal WPRE). The PGK promoter was replaced with an SFFV promoter PCR-amplified from pHRSIN-cPPT-SEW to generate pHRSIREN-S-SBP-ΔLNGFR-W. BamHI and EcoRI sites allow insertion of alternative shRNAs of interest into the U6-shRNA cassette as described for the pSIREN-RetroQ vector (Clontech).

For CRISPR/Cas9 genome editing, P2A-SBP-ΔLNGFR was subcloned from pHRSIN-SE-P2A-SBP-ΔLNGFR-W into pSpCas9(BB)-2A-Puro (PX459; Addgene) to generate pSpCas9(BB)-P2A-SBP-ΔLNGFR (encoding a U6-guide RNA (gRNA) cassette and human codon-optimized *S. pyogenes* Cas9 (SpCas9) co-translated with SBP-ΔLNGFR via a P2A peptide linker). BbsI sites allow insertion of site-specific gRNAs identified using the CRISPR Design Tool (http://crispr.mit.edu) [17] according to protocols kindly made available by Feng Zhang (http://www.genome-engineering.org) [18].

The final nucleotide and amino acid sequences of the codon-optimised SBP-ΔLNGFR construct are shown (Sequence S1). For knockdown of β2-microglobulin (β2 m), the following shRNA target sequence was used: 5′-GAATGGAGAGAGAATTGAA-3′ [12]. For knockout of β2 m, the following gRNA target sequence was kindly selected and subcloned by Dick van den Boomen: 5′-GGCCGAGATGTCTCGCTCCG-3′. For functional assays in primary human CD4+ T cells, the following control (non-targeting) shRNA sequence was kindly provided by Greg Towers: 5′-GTTATAGGCTCGCAAAAGG-3′.

### Transfection and lentiviral transduction

FuGENE 6 (Promega; lentiviral production) or TransIT-293 (Mirus; general transfections) were used for plasmid DNA transfections in 293Ts. To generate pseudotyped lentiviral stocks, 293Ts were co-transfected with pHRSIN−/pHRSIREN-based lentivector, pCMVR8.91 and pMD.G, media changed at 24 hrs and viral supernatant harvested and filtered (0.45 µm) at 48 hrs prior to concentration using Lenti-X Concentrator (Clontech) or storage at –80°C. Transduction of primary human CD4+ T cells 6–24 hrs after activation was performed by spinoculation at 800 g for 1–2 hrs in a benchtop centrifuge.

### Flow cytometry

293Ts were harvested with enzyme-free cell dissociation buffer and Dynabeads Human T-Activator CD3/CD28 beads were removed from primary human CD4+ T cells using a DynaMag-2 magnet (Invitrogen). Typically 2×10^5^ washed cells were incubated for 30 mins in 100 µL PBS with the indicated fluorochrome-conjugated antibody or streptavidin-APC. All steps were performed on ice or at 4°C and stained cells were analysed immediately or fixed in PBS/1% paraformaldehyde. Viability was assessed using forward and side scatter, and absolute cell numbers determined using CytoCount beads (Dako) as a reference population according to the manufacturer’s instructions.

### Antibody-free magnetic cell sorting

For pilot experiments in transfected 293Ts (Figure 1), washed cells were harvested with enzyme-free dissociation buffer and filtered (50 µm) to remove clumps. For selection using Dynabeads Biotin Binder (Invitrogen), cells were resuspended in Incubation Buffer (IB; PBS without calcium/magnesium, 2 mM EDTA, 0.1% BSA) at 10^7^ cells/ml and incubated with Dynabeads at a bead-to-total cell ratio of 4∶1 for 30 mins at 4°C. Bead-bound cells were selected using a DynaMag-2 (Invitrogen) then released from the beads by incubation in IB supplemented with 2 mM biotin for 15 mins at room temperature (RT) and analysed by flow cytometry. For selection using Streptavidin MicroBeads (Miltenyi) cells were resuspended in IB at 2.5×10^7^ cells/ml and incubated with MicroBeads at a bead-to-total cell ratio of 10 µl:10^7^ cells for 30 mins at 4°C. Bead-bound cells were selected using an MS Column and MACS Separator (Miltenyi) and analysed by flow cytometry without MicroBead removal. For selection of transduced primary human CD4+ T cells, Dynabeads Human T-Activator CD3/CD28 beads were first removed according to the manufacturer’s instructions. An optimised protocol for Antibody-Free Magnetic Cell Sorting using Dynabeads Biotin Binder is supplied (Protocol S1). For functional assays in primary human CD4+ T cells, mock (unselected) cells were subjected to a sham selection procedure of equivalent duration, including incubation with Dynabeads Biotin Binder.

## Results and Discussion

### The 38 amino acid SBP may be displayed at the cell surface by fusion with the truncated LNGFR

The 38 amino acid SBP is a high-affinity streptavidin-binding peptide tag previously used for purification of recombinant proteins and, more recently, as an affinity tag in live cells for the synchronisation of secretory traffic [10], [11], [19]. To express the 38 amino acid SBP at the cell surface, we fused it to the N-terminus of the truncated LNGFR (SBP-ΔLNGFR; Figure 1B). 293Ts transfected with this construct were readily stained with streptavidin-APC in the absence of permeabilisation, indicating expression of SBP-ΔLNGFR at the plasma membrane and accessibility for streptavidin binding (Figure 1C). SBP-ΔLNGFR was also readily detected using an LNGFR-specific antibody (Figure 1D). LNGFR is a 399 amino acid Type I transmembrane cell surface glycoprotein member of the Tumour Necrosis Factor Receptor superfamily [20]. The truncated LNGFR, which lacks a cytoplasmic domain, has been previously used as a non-functional cell surface marker for antibody-based cell selection, including *in vitro* and *in vivo* for purification of transduced human lymphocytes in the setting of allogeneic bone marrow transplantation [21], [22]. The level of cell surface streptavidin-binding peptide expression achieved was critically dependent on the fusion protein chosen, since preliminary experiments using the 38 amino acid SBP fused to the HLA-A2 heavy chain, or the streptavidin-binding Nano-tag peptide fused to a membrane-targeted red fluorescent protein construct [9], [23], showed poor staining at the surface of transfected cells.

### Cells expressing SBP-ΔLNGFR may be selected using streptavidin-conjugated magnetic beads

To test whether SBP-ΔLNGFR could be used for cell selection, we incubated transfected 293Ts with streptavidin-conjugated magnetic beads. Bead-bound cells were washed, and then either analysed directly by flow cytometry, or released from the beads by incubation with excess biotin. Selected cells were markedly enriched for SBP-ΔLNGFR expression, and comparable results were achieved using streptavidin-conjugated beads from 2 different manufacturers (Figure 1D). Dynabeads Biotin Binder were used for subsequent experiments at an optimised bead-to-target cell ratio of 10∶1. Although the 38 amino acid SBP interacts strongly with streptavidin (nanomolar Kd, comparable to a strong antibody-antigen interaction), it is readily out-competed by biotin (femtomolar Kd, one of the strongest non-covalent interactions known) [10], [19], [24], [25]. In practice, bound cells could be completely released from streptavidin-conjugated beads by incubation with 2 mM biotin for as little as 15 mins. Magnetic selection of cells expressing cell surface streptavidin (using bead-bound anti-streptavidin antibody) or co-expressing a cell surface biotin-acceptor peptide with the *E. coli* biotin ligase BirA (using streptavidin-conjugated beads) has been previously described [26], [27], [28], as has FACS of cells expressing a cell surface biotin-mimetic peptide (using fluorochrome-conjugated streptavidin) [29]. Conversely, this is the first report of the use of a cell surface streptavidin binding peptide for magnetic cell sorting, combining the advantages of bead-based cell isolation with the ability to release beads from selected cells by competition with biotin.

### SBP-ΔLNGFR affinity purification may be used to isolate cells expressing an shRNA or exogenous gene of interest

To select genetically modified mammalian cells using SBP-ΔLNGFR affinity purification, we co-expressed the fusion protein with an exogenous gene or shRNA on the same lentiviral construct. As proof of principle, SBP-ΔLNGFR was subcloned into lentiviral vectors encoding either GFP or an shRNA to β2-microbglobulin (β2 m). β2 m is an essential subunit of MHC class I molecules and its depletion may therefore be detected by reduction of cell surface MHC class I alleles such as HLA-A2 [30]. Co-expression of SBP-ΔLNGFR with GFP (Figure 2A) or shRNA to β2 m (Figure 2B) was confirmed by transient transfection of 293Ts, and similar results were obtained using VSVg-pseudotyped lentiviral particles (Figures 2A and 2B). The selection of cells genetically modified *ex vivo* remains a significant methodological challenge for human gene therapy. As well as the treatment of monogenic disorders such as ADA-SCID (adenosine deaminase deficiency resulting in severe combined immunodeficiency) major research efforts have focussed on cancer immunotherapy using engineered T cells expressing tumour-specific T cell receptor α and β chains (αβTCRs) or chimeric antigen receptors (CARs), and the production of HIV-resistant CD4+ T cells through, for example, disruption or downregulation of the CCR5 HIV co-receptor [31], [32], [33]. We therefore decided to test whether magnetic selection for SBP-ΔLNGFR could be used to purify genetically modified primary human CD4+ T lymphocytes expressing an exogenous gene or shRNA of interest. Indeed, following lentiviral transduction and SBP-ΔLNGFR affinity purification, pure populations of cells either high in GFP or low in HLA-A2 were successfully isolated (Figures 2C and 2D).

### Antibody-free magnetic cell sorting yields >99% pure populations of primary human CD4+ T cells in <1 hr

Expression of SBP-ΔLNGFR from the PGK promoter was noted to vary markedly according to the activation state of transduced T cells (Figure 3A). PGK encodes the glycolytic enzyme phoshpoglycerokinase, and glycolysis is known to be highly regulated in T cells [34]. To optimise the system for selecting primary human lymphocytes, we therefore introduced the SFFV promoter to drive expression of SBP-ΔLNGFR either as a single cistron (pHRSIREN-S-SBP-ΔLNGFR-W) or co-translated with an exogenous gene of interest via a P2A “self-cleaving” peptide linker for bicistronic expression (pHRSIN-SE-P2A-SBP-ΔLNGFR-W). These modifications increased SBP-ΔLNGFR expression without compromising levels of the co-expressed gene or shRNA of interest (Figure 3B). Expression levels were critically dependent on both the WPRE and the promoter strategy used, with inferior results obtained using the EF1a promoter, ECMV IRES or dual SFFV promoter systems, or when the WPRE was absent or alternatively located. The SFFV promoter is known to provide high-level transgene expression in primary human haematopoietic cells [14] and 2A peptides have been shown to enable stoichiometric co-expression of multiple cistrons across different organisms and cell types [15], [35]. These small viral peptide sequences are co-translationally “cleaved” in a process known as “ribosomal skipping” in which formation of a glycyl-prolyl peptide bond at the C-terminus of the 2A peptide is “skipped” without interrupting translation of the downstream polypeptide [36]. To test the optimised vectors (Figure 3C), we transduced primary human CD4+ T cells using 4 different constructs (expressing 2 different shRNAs and 2 different exogenous genes). From a starting purity of 31.0%, the average purity of selected cells was 99.2% (Figure 3D). We have not observed any functional deficits in a wide range of downstream applications (including viability, expression of activation markers, and proliferation; Figure 3E), and the Antibody-Free Magnetic Cell Sorting procedure (from incubation with magnetic beads through release with biotin) may be readily completed (including multiple samples) in <1 hr.

### Antibody-free magnetic cell sorting allows isolation of cells following CRISPR/Cas9 genome editing

The type II bacterial CRISPR “immune system” has recently been re-purposed to allow facile site-specific genome engineering in mammalian cells by co-expression of the Cas9 nuclease with a short gRNA [18], [37], [38]. Complementary base-pairing through the gRNA recruits the gRNA/Cas9 complex to a target sequence in the genomic DNA, where it introduces double-strand DNA breaks. Where the target sequence is in an open reading frame (ORF), repair of these breaks by non-homologous end joining frequently introduces short insertions and deletions (InDels), leading to frameshifts and/or premature stop codons and knockout of the targeted gene. Alternatively, if an exogenous DNA repair template is also supplied, homology-directed repair may be utilised to copy this template to the cut target sequence, allowing the introduction of specific nucleotide changes or “knock-in” of exogenous sequences. As compared with the requirement for persistent expression of an shRNA or exogenous gene of interest, an advantage of CRISPR/Cas9 genome editing is that transient expression of the Cas9 nuclease and site-specific gRNA plus/minus DNA repair template may be used to introduce permanent genetic modifications. Conversely, this approach makes selection of genetically modified cells using antibiotics or FACS challenging, since expression from the CRISPR/Cas9 vectors is time-limited. To test whether Antibody-Free Magnetic Cell Sorting could be used to isolate cells following CRISPR/Cas9 genome editing, we transfected 293Ts with a vector encoding a gRNA targeting the 5′ end of the β2 m gene and SBP-ΔLNGFR co-translated with Cas9 via a P2A peptide linker (Figure 4A). As with shRNA knockdown, knockout of the β2 m gene may be detected by reduction in cell surface MHC class I (MHC-I). Following Antibody-Free Magnetic Cell Sorting, MHC-I low cells were markedly enriched (Figure 4B). The ability to enrich genetically modified cells may be particularly helpful if the phenotype associated with the genetic modification is either unknown or unsuitable for subsequent selection by FACS, and/or in situations where difficult-to-transfect cells are used, resulting in a low frequency of cells with the desired genetic modification and the necessity to screen multitudinous clones.

## Conclusions

Antibody-Free Magnetic Cell Sorting is a novel, efficient way to select transfected or transduced mammalian cells. Selection is readily scalable to almost any cell number and may be completed in <1 hr (plus cell washes). No antibody is required, allowing rapid one-step affinity purification and making the process extremely cost-effective. Enrichment to >99% purity is routinely achieved and, following release with biotin, cells are left “untouched” by residual beads or antibody-antigen complexes. As well as providing a useful tool for life sciences research, the system may be used to select genetically modified cells for human gene therapy applications. Potential applications of Antibody-Free Magnetic Cell Sorting are not limited to the expression of shRNAs or exogenous genes of interest, and CRISPR/Cas9 genome editing. For example, we are currently developing vectors for one-step magnetic selection of cells following infection with an HIV reporter virus [39], and expression of SBP-ΔLNGFR may be used as a reporter gene for selection of cells in which a promoter of interest is active *in vitro* or *in vivo*.

## Supporting Information

Protocol S1 Antibody-Free Magnetic Cell Sorting.
(PDF)

Sequence S1 Codon-optimised SBP-ΔLNGFR construct.
(PDF)
