# Supplementary material for: Antibody-Free Magnetic Cell Sorting of Genetically Modified Primary Human CD4+ T Cells by One-Step Streptavidin Affinity Purification
Source: PLoS One. 2014 Oct 31;9(10):e111437. doi: 10.1371/journal.pone.0111437 (PMC4216076; doi:10.1371/journal.pone.0111437)
Supplement: Protocol S1 — Antibody-Free Magnetic Cell Sorting. (PDF) [file pone.0111437.s001.pdf]

Protocol S1. Antibody-Free Magnetic Cell Sorting

The following protocol has been optimised for Antibody-Free Magnetic Cell Sorting of transduced primary human CD4+ T cells to maximum purity using Dynabeads Biotin Binder.

It may be readily scaled for any cell number and minimally-adapted for other transfected or transduced cell types.

- Adherent cells **must** be harvested with enzyme-free dissociation buffer
- All cells **must** be washed thoroughly to avoid carry-over of biotin from culture media
- Where indicated by the manufacturer, streptavidin-conjugated beads **must** be washed before use to remove preservative and/or free (unconjugated) streptavidin

Incubation Buffer (IB)<br>Pre-cool on ice	PBS without calcium/magnesium, pH 7.4<br>2mM EDTA<br>0.1% BSA (A4503; Sigma)
Release Buffer (RB)<br>Pre-warm to 37°C	Complete media e.g. RPMI-1640 with 10% FCS and 1% pencillin/streptomycin<br>10mM HEPES buffer, pH 7.4<br>2mM biotin

1. If required remove Dynabeads Human T-Activator CD3/CD28 beads (Invitrogen) according to the manufacturer's instructions.

2. Wash cells 3x with cold IB then resuspend in same at 10^7 cells/ml.

3. Add Dynabeads Biotin Binder at a bead:transduced cell ratio of 10:1 and incubate at 4°C for 30 mins with gentle agitation

4. Place tube on appropriate magnet for 2-3 mins and remove supernatant containing unbound cells.

5. Gently wash bead-bound cells 1-2x with cold IB then return to magnet for 2-3 mins and remove supernatant containing unbound cells.

6. Resuspend bead-bound cells in pre-warmed RB at no more than 10^7 cells/ml and incubate at room temperature for 15 mins with gentle agitation.

7. Place tube on appropriate magnet for 2-3 mins then transfer supernatant containing released cells to new tube.

8. If desired, to maximise yield, wash beads 1x with RB then return to magnet for 2-3 mins and pool supernatants containing released cells.

9. Wash released cells 2x with complete media and use as required for downstream applications.
