# Supplementary material for: Antibody-Free Magnetic Cell Sorting of Genetically Modified Primary Human CD4+ T Cells by One-Step Streptavidin Affinity Purification
Source: PLoS One. 2014 Oct 31;9(10):e111437. doi: 10.1371/journal.pone.0111437 (PMC4216076; doi:10.1371/journal.pone.0111437)
Supplement: Sequence S1 — Codon-optimised SBP-ΔLNGFR construct. (PDF) [file pone.0111437.s002.pdf]

Figure S1. Codon-optimised SBP-ΔLNGFR construct

DNA and amino acid sequences of the codon-optimised SBP-ΔLNGFR construct in pHRSIN-SE-P2A-SBP-ΔLNGFR-W are shown. BamHI and NotI sites may be used to insert the gene of interest (without stop codon) upstream of the P2A peptide for co-translation with SBP-ΔLNGFR. In pHRSIREN-S-SBP-ΔLNGFR-W, the coding sequence starts with the murine immunoglobuin signal peptide (*) and the shRNA of interest is inserted separately in the U6-shRNA cassette using BamHI and EcoRI sites. In pSpCas9(BB)-P2A-SBP-ΔLNGFR the Cas9 nuclease is sited upstream of the P2A peptide for co-translation with SBP-ΔLNGFR and the gRNA of interest is inserted separately in the U6-gRNA cassette using BbsI sites. Locations of ribosomal skipping (†) and signal peptidase (‡) cleavage are shown. Following signal peptidase cleavage the construct is anchored to the plasma membrane by the transmembrane region (TM) of the truncated LNGFR. P2A peptide – blue; signal peptide – tan; SBP – red; ΔLNGFR – grey and dark grey (TM region). Unshaded amino acids comprise flexible linker regions. Assembly is modular and unique restriction sites are highlighted.

```
NotI
gcg gcc gca gga agc gga gct act aac ttc agc ctg ctg aag cag gct gga gac gtg gag
 A   A   A   G   S   G   A   T   N   F   S   L   L   K   Q   A   G   D   V   E
                        P2A peptide

             †       *
gag aac cct gga cct atg ggc tgg tca tgt atc att ctg ttt ctg gtc gca acc gca act
 E   N   P   G   P   M   G   W   S   C   I   I   L   F   L   V   A   T   A   T
                    Murine immunoglobulin signal peptide

                            XhoI
gga gtg cat tca cag gtg cag ctc gag ggg tca ggg atg gac gaa aag acc aca gga tgg
 G   V   H   S   Q   V   Q   L   E   G   S   G   M   D   E   K   T   T   G   W
             ‡                                  38 amino acid SBP

cga gga gga cac gtg gtc gag gga ctg gca gga gag ctg gaa cag ctg cgg gct aga ctg
 R   G   G   H   V   V   E   G   L   A   G   E   L   E   Q   L   R   A   R   L

                                                    AsiSI
gaa cac cat cct cag gga cag cga gag cca gga agt gga gcg atc gcg aag gaa gcc tgc
 E   H   H   P   Q   G   Q   R   E   P   G   S   G   A   I   A   K   E   A   C
                                                                ΔLNGFR

ccc aca ggg ctg tac act cat tct gga gaa tgc tgt aaa gct tgt aac ctg gga gag gga
 P   T   G   L   Y   T   H   S   G   E   C   C   K   A   C   N   L   G   E   G

gtg gca cag cca tgc gga gcc aat cag act gtg tgc gag cct tgt ctg gac tcc gtc aca
 V   A   Q   P   C   G   A   N   Q   T   V   C   E   P   C   L   D   S   V   T

ttc tct gat gtg gtc agt gcc aca gaa cct tgc aag cca tgt act gag tgc gtg ggc ctg
 F   S   D   V   V   S   A   T   E   P   C   K   P   C   T   E   C   V   G   L

cag tct atg agt gct cct tgt gtg gag gct gac gat gca gtc tgc cgg tgt gca tac gga
 Q   S   M   S   A   P   C   V   E   A   D   D   A   V   C   R   C   A   Y   G

tac tat cag gac gag act acc ggc aga tgt gaa gct tgc agg gtg tgt gag gca ggc tca
 Y   Y   Q   D   E   T   T   G   R   C   E   A   C   R   V   C   E   A   G   S

ggg ctg gtc ttt agc tgc cag gat aaa cag aac acc gtg tgc gag gaa tgt cct gac ggg
 G   L   V   F   S   C   Q   D   K   Q   N   T   V   C   E   E   C   P   D   G

aca tat agc gat gag gcc aat cac gtg gac ccc tgc ctg cct tgt act gtg tgc gag gat
 T   Y   S   D   E   A   N   H   V   D   P   C   L   P   C   T   V   C   E   D

acc gaa agg cag ctg cgc gaa tgt acc aga tgg gca gac gcc gag tgc gag gaa atc cca
 T   E   R   Q   L   R   E   C   T   R   W   A   D   A   E   C   E   E   I   P

ggg cga tgg att act cgg tcc acc ccc cct gaa gga tca gac agc acc gca cca tct aca
 G   R   W   I   T   R   S   T   P   P   E   G   S   D   S   T   A   P   S   T

cag gag cca gaa gca cca cca gag cag gat ctg atc gcc tcc acc gtg gct ggc gtg gtc
 Q   E   P   E   A   P   P   E   Q   D   L   I   A   S   T   V   A   G   V   V

aca act gtc atg ggg agc tcc cag cca gtg gtc acc cgg ggc acc aca gat aac ctg att
 T   T   V   M   G   S   S   Q   P   V   V   T   R   G   T   T   D   N   L   I
                                                                        TM

ccc gtg tat tgc tcc atc ctg gca gcc gtc gtc gtc gga ctg gtg gca tac atc gcc ttc
 P   V   Y   C   S   I   L   A   A   V   V   V   G   L   V   A   Y   I   A   F

            KpnI
aag cgg tga ggt acc
 K   R   -
```
